# Real-time mode of operation data analysis to catch the thread-tip denotes the failure cause of the grid-tie PV central inverter

**DOI:** 10.1038/s41598-023-41520-8

**Published:** 2023-09-08

**Authors:** Youssef Badry Hassan, Mohamed Orabi, Mahmoud A. Gaafar

**Affiliations:** Aswan Power Electronic Applications Research Center (APEARC), Aswan University, Aswan, 81542 Egypt

**Keywords:** Engineering, Electronics, photonics and device physics

## Abstract

The inverter is considered the core of the PV power plant. The inverter’s failure leads to generation loss and decreases plant availability. So, it is required to investigate a clear Root Cause Analysis (RCA) to deduce the failure causes and implement the required corrective action in addition to the preventive action to avoid more inverter failure, hereby maintaining the plant available to a certain value. This paper discusses real-time mode operation data analysis of the PV grid-connected inverter due to real central inverter incidents in Benban solar park located in Egypt.The central inverter plays an important role in the Mega-Scale PV power plant. The main function of this inverter is to convert the DC power produced by the PV modules to AC power to be injected into the utility grid by considering specific characteristics based on the grid code. The availability of any PV power plant directly depends on the healthy inverter’s operation. The more increases for the installed inverters, the less availability loss in the case of inverter partial or catastrophic failures. So, it is required to focus on the failure causes of the central inverter by implementing a technical analysis using the available operational data. The monitored data of the central inverter in the PV power plant is classified into two types. The first type is the continuous time data stored in the memory. It represents the waveforms of inverter outputs like voltage, current, frequency, …. etc. Unfortunately, in case of a catastrophic failure, the central inverter is completely charred, and the continuous time data is lost due to storage memory damage. The second type is the operation data that is recorded by the SCADA system (per one-minute interval). Hereby, the operation data is the sole data in the case of the completely charred inverter. The representation of the operational data in curves indicates symptoms that can be used for the RCA processes. The investigation outcomes include three results. The first result is detecting the signature of the IGBT thermal stress on the voltage balance of the DC link capacitor. The second result is verifying a scenario for the cause of the IGBT failure by implementing a technical mathematical model based on the detected symptoms that denote the fault signature which is considered the thread-tip for detecting the failure cause. The third result is the simulating scenario for the interpretation of a DC link capacitors explosion due to the short circuit fault that occurred due to IGBT failure. The investigation in this paper is performed based on operation data analysis of the PV grid-connected inverter (central type) due to a real incident. The analysis methodology is based on mathematical calculation for the IGBT junction temperature using the measured heatsink temperature. The study concludes that after the IGBT failure occurred, it was a short circuit for a while and closed the terminals of the DC link capacitors. So, the DC link capacitors exploded and produced heavy sparks that led to enough fire to burn the inverter container completely.

## Introduction

To mitigate the climatic changes, most countries search for renewable energy sources for electrical energy generation to replace conventional electrical power plants that use fossil fuels. Limiting fossil fuel consumption leads to decreasing the impact of global warming phenomena^1–3^. So, most countries start the installation of wind farms and PV power plants to generate electrical energy^4^. In addition to encouraging electricity consumers to apply energy saving and install off-grid PV systems, especially for irrigation and remote loads. Also, recycling wastes to mitigate the environmental impact^5–11^. Mega-Scale PV power plants are widely installed to generate green electrical energy. So, many investors are interested in investing more money to construct Mega-Scale PV power plants. Then many experts and research and development establishments focus on performance improvements and avoiding the malfunctions which lead to business interruption in the PV power plants^12^. The large-scale PV power plant consists of two main parts. The first part includes the DC components, while the second part includes the AC components^13,14^. The inverter is considered the core of this large-scale PV power plant that is used to convert the DC power produced by many strings of the PV modules to AC power which is injected into the grid. The availability of a PV power plant is measured based on the inverter’s working hours^15^. If any inverter failure occurs, the availability decreases. To maintain maximum availability, it is required to maintain the PV inverter runs without intermittent during sunlight hours. So, it is required to focus on the PV inverter performance and study the failure reasons to implement both corrective and preventive actions^16^.

As mentioned in^17^, the previous research publications discussed the inverter RCA from the laboratory stage as initiating some faults such as open circuit faults or short circuit faults. The other previous publications discussed the technical investigation by the visual inspection defects, then deduce some failure causes that may be correct or not correct as there is no verification or validation model. Then a new methodology is investigated to find the failure case analysis of the PV grid-tie inverter.

Different types of IGBT failures are discussed and reviewed in^18^ which are summarized as the following:

- Bond wire fatigue.
- Soldering fatigue.
- Deformation of metallization.
- Corrosion of bond wire and metallic connections.
- Semiconductor cracks.

Benban solar park locates in Aswan governorate, the southernmost part of Egypt. It includes 32 large-scale PV power plants. Some of these PV plants have a capacity of 50 MW of AC power per each, and others have a capacity of 25 MW of AC power per each. The total capacity of Benban solar park is 1600 MW of AC power. In Benban solar park, the central inverter is commonly used to convert the DC power produced by many strings of the PV modules to AC power which is injected into the grid. There have been many inverters’ incidents. This paper presents an analytical model of the inverter operational data before fault occurrence.

## Method

**Figure 1:**
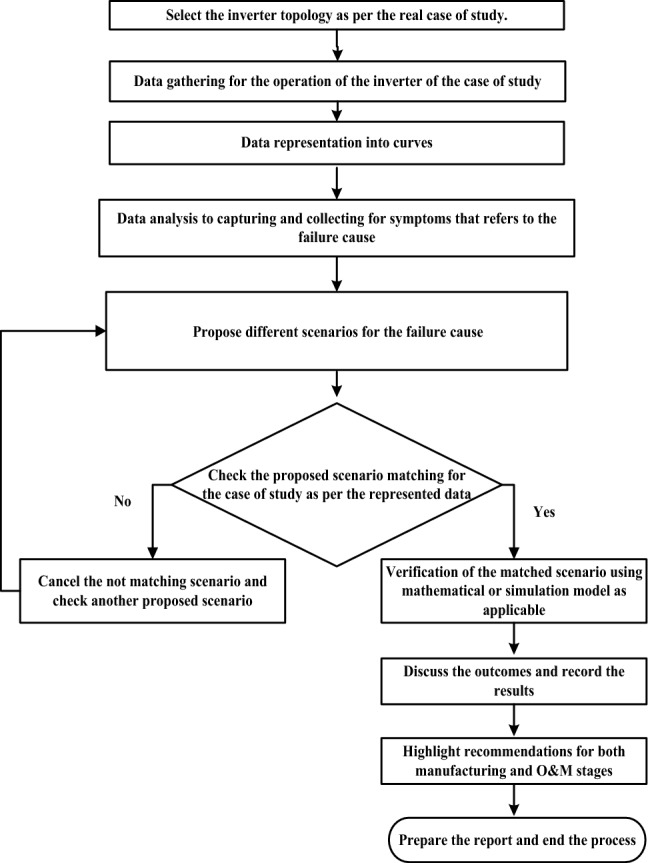
Flowchart of FCA methodology for PV grid-tie inverter.

The investigated work in this paper considers the initial implementation of the investigated methodology in^17^ as summarized in the flowchart in Figure 1.

This research method aims to deduce the IGBT junction temperature from the measured heatsink temperature to indicate the IGBT thermal stress level.

The setting of the study process starts with data gathering, then data representation to capture symptoms indicate the fault signatures which consider the thread-tip denotes the failure cause. The data processing is performed through the mathematical model to deduce the unmeasured parameters. The expected scenarios for the failure shall be verified and approved to deduce a real RCA. In addition to detecting the signature of the IGBT thermal stress on the voltage balance of the DC link capacitor. Further, interpretation for DC link capacitors explosion. The investigation is performed for a 1500 kW PV inverter based on real operational data. It is divided into several stages that are discussed in the following sections.

### Overview of the studied inverter topology

**Figure 2:**
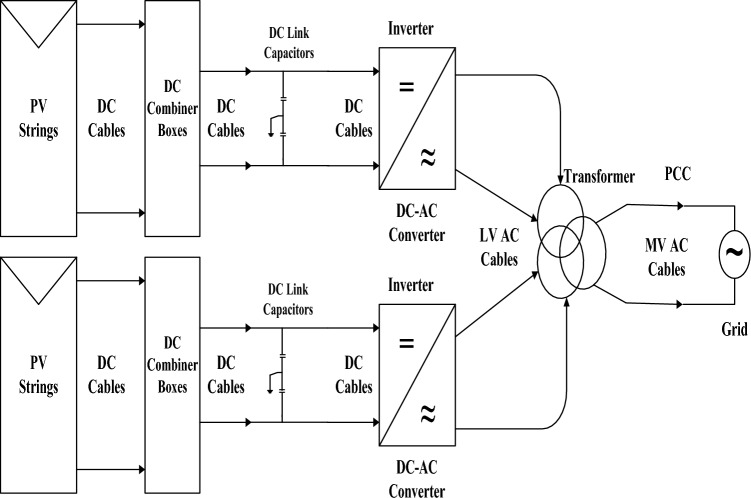
SLD of DC components in the PV plant^19^.

The inverter components are classified into three sections as shown in Figure 2. The first type belongs to the DC part such as the DC link capacitor and the DC circuit breakers. The second section belongs to the AC part such as the harmonic filter, AC circuit breakers, and cooling fans. The third section is the transition part that converts the DC power to AC power. It consists of power electronic switches and multi-function PCBs that are used to control, protect, and monitor the inverter operation^19^. In this case, the MPPT is considered in the inverter control system.

As mentioned in^20–22^, the power losses of three-level inverters are greater than that for the two-level inverters due to the larger number of power electronics switches and capacitors of the DC link. Hereby, the inverter efficiency is higher for two-level inverters than for three-level inverters.

As mentioned in^22,23^, the Total Harmonic Distortion (THD) for the three-level inverter is lower than that for the two-level inverter due to the larger number of power electronics switches.

**Figure 3:**
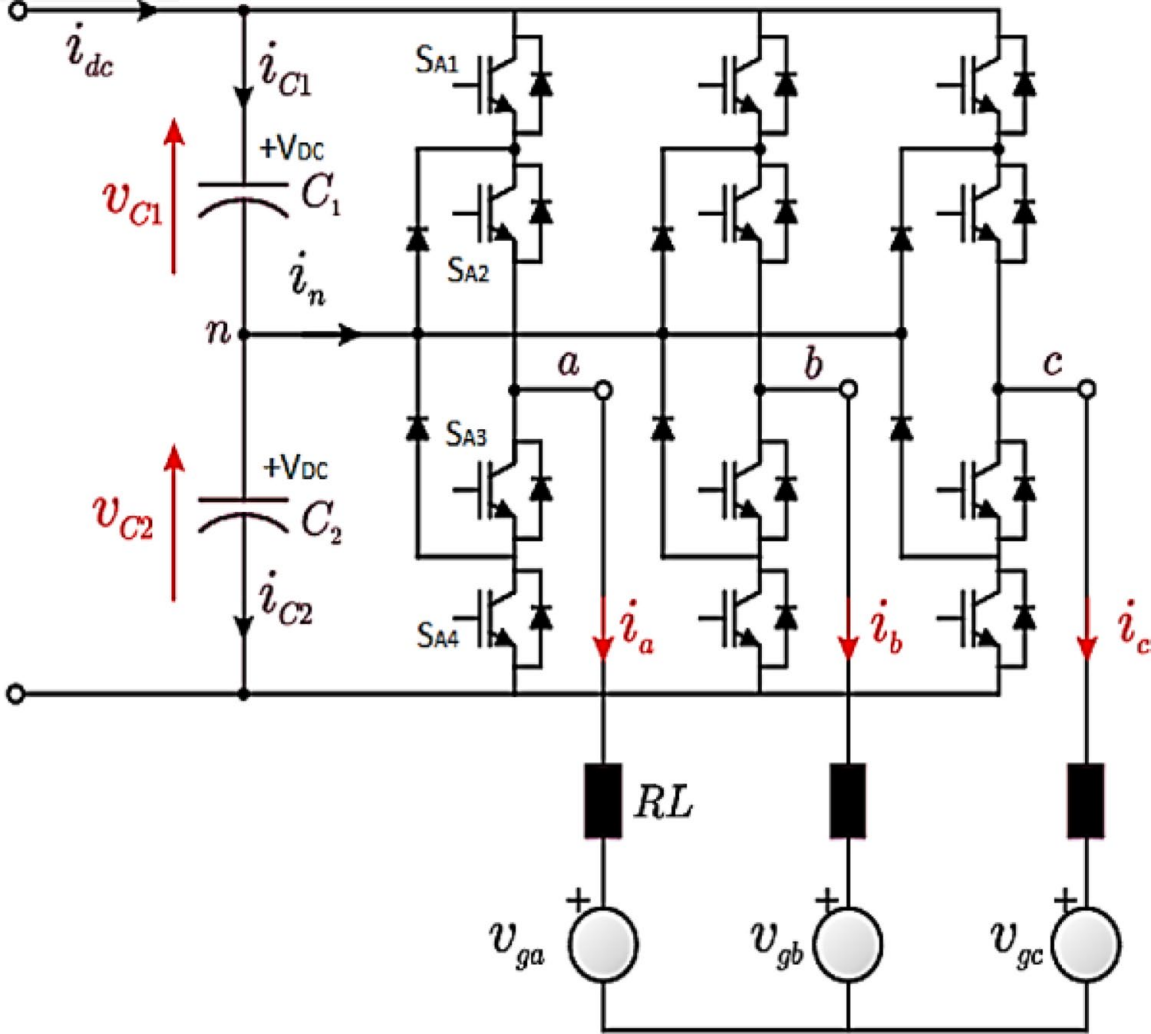
Five level—three phase neutral point clamped inverter^24^.

**Figure 4:**
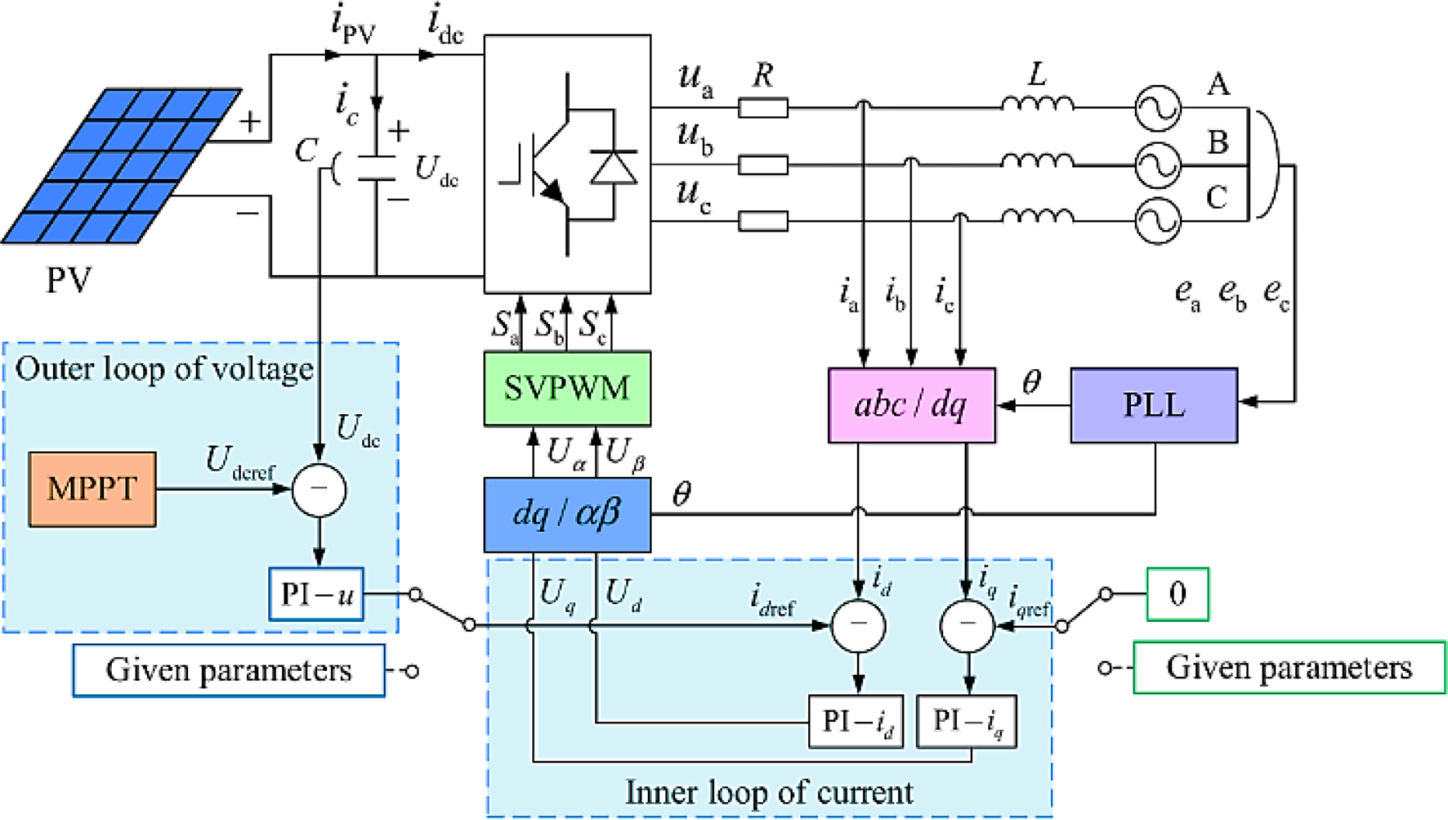
Decoupling closed-loop control for the PV grid-tie inverter based on PI compensation^25^.

As in the Mega-scale PV power plants, the grid requirement for the THD is very low and shall not exceed 5%, also the power losses due to the power electronic switches are minor or very low compared with the total rating capacity of the PV grid-tie inverter which is rated in Mega-watt. The real case which is investigated in this paper is a three-level NPC inverter. The inverter power circuit consists of three legs as shown in Fig. 3^24^. The control system for that inverter is shown in Fig. 4, it consists of two closed loops as follows^25–27^:

- The inner loop to control the inverter current.
- The outer loop to control and regulate the DC-link capacitor voltage to the Maximum Power Point Tracking (MPPT) of the PV strings.

### Data gathering

A brainstorming session was conducted with the O&M team to collect more information about the inverter incident that may be helpful for this analysis. The session outcomes are as the following:

- The average wind speed on the incident day was 4.5 m per second and there was no dust or sand storming. So, the air filter didn’t suffer from heavy dust or sand accumulation.
- Semi-cloudy weather was observed for many intervals during the incident day as confirmed by the radiation curves.
- The weather in Benban solar park is dry. Therefore, the air humidity does not affect the operating conditions of the inverter.
- The site technician observed fire and smoke emitted from the inverter container, as well as successive popping sounds due to exploding DC bus capacitors. Therefore, a short circuit path is created on the terminals of the DC bus capacitors. It is required to find the cause of this short circuit fault.
- A visual inspection of the burnt inverter debris confirmed that there was nothing to be inferred, as all inverter components were charred.
- Because the storage memory board was completely burnt, the design data was lost.

In this plant, the DC section which is input to each inverter consists of multiple strings which are connected in parallel as shown in Fig. 2. Each inverter is fed by 24 combiner boxes. Each combiner box consists of 12 PV strings. Each string c of 30 PV modules.

The DC parameters include the following:

- The total DC strings voltage (V_DC_).
- The total DC current (I_DC_).
- The total DC Power (P_DC_).

The AC parameters include the following:

- The phase AC current (I_a_, I_b_, I_c_).
- The line-to-line AC voltage (V_ab_, V_bc_, V_ca_).
- The AC active power (P_AC_).
- The AC reactive power (Q).
- The AC apparent power (S).
- The power factor (PF).

The other monitored inverter parameters include the following:

- The inverter cabinet temperature (T__Cab_). It refers to the temperature inside the inverter container.
- The IGBT heatsink temperature (T__HS_).

The weather station parameters include the following:

- The ambient temperature (T_Amb_).
- The horizontal global irradiance (GHI).
- The tilted global irradiance (GTI).
- The wind speed (Ws).

As the operational data measured only the heatsink temperature, it is required to calculate the IGBT junction temperature. The next section focuses on the calculation of the IGBT power losses to calculate the junction temperature.

### The mathematical deduction of IGBT junction temperature

**Figure 5:**
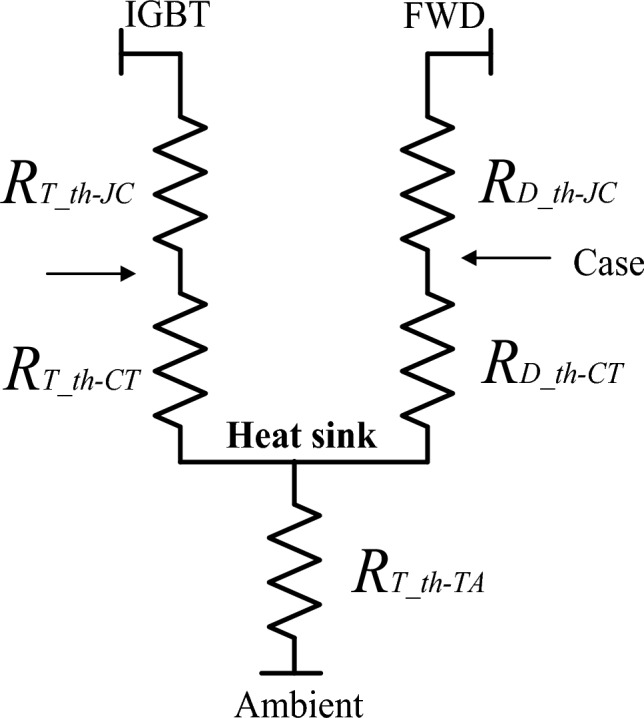
The thermal network representation for the IGBT and its body diode in one chip.

To calculate IGBT junction temperature, it is required to calculate the total power dissipation which divides into conduction power loss as given in Eq. (1), in addition to switching power loss as given in Eq. (2). Then, the IGBT power loss is calculated as given in Eq. (3). The thermal network for each IGBT built with FWD in the same body is shown in Fig. 5^28,29^.

1 $${P}_{IGBT\_Cond}={V}_{CE}{I}_{IGBT\_av}+{I}_{IGBT\_rms}^{2}{r}_{CE}$$

2 $${P}_{IGBT\_Sw}=(\frac{{K}_{g}{f}_{s}}{\pi })(\frac{{V}_{\mathrm{op}}{I}_{\mathrm{op}}}{{V}_{Nom}{I}_{Nom}})({E}_{\mathrm{on}}+{E}_{off})$$

3 $${P}_{IGBT\_Loss}={P}_{IGBT\_Cond}+{P}_{IGBT\_Switching}$$

The IGBT junction temperature is given by Eq. (4).

4 $${T}_{J\_IGBT}={T}_{Cas}+{P}_{IGBT\_Loss}.{R}_{eq\_th(J-Cas)}$$

All the parameters are given for the proposed case as per SIMKRON datasheet (SKM1200MLI12BE4_22898514 & SKM1200MLI12TE4_22898513) for the used IGBT which is available online.

### The operational data representation of the last period before the inverter incident

**Figure 6:**
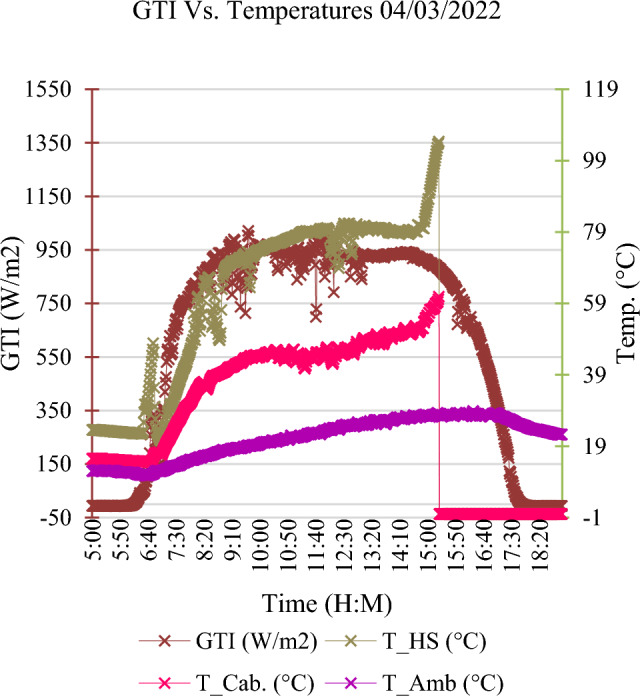
A real profile (irradiance & temperature) for the studied case.

A real profile (irradiation and temperature) is shown in Fig. 6, the temperature of the IGBT increased from 73 to 81 °C during the period from 12:00 PM to 02:20 PM due to radiation increase, in addition to both ambient and cabinet temperatures increased. In the time of 02:54 PM, the heat sink temperature increased gradually until it reached 104°C at the time of failure. This indicates to a malfunction of the cooling fan happened. The healthy and faulty operation of the cooling fan is monitored by the SCADA system. This is implemented via the feedback of the contactor open that indicates to start or stop of the cooling fan. So, if the malfunction is located after the contactor, it can’t be discovered by the SCADA system.

**Figure 7:**
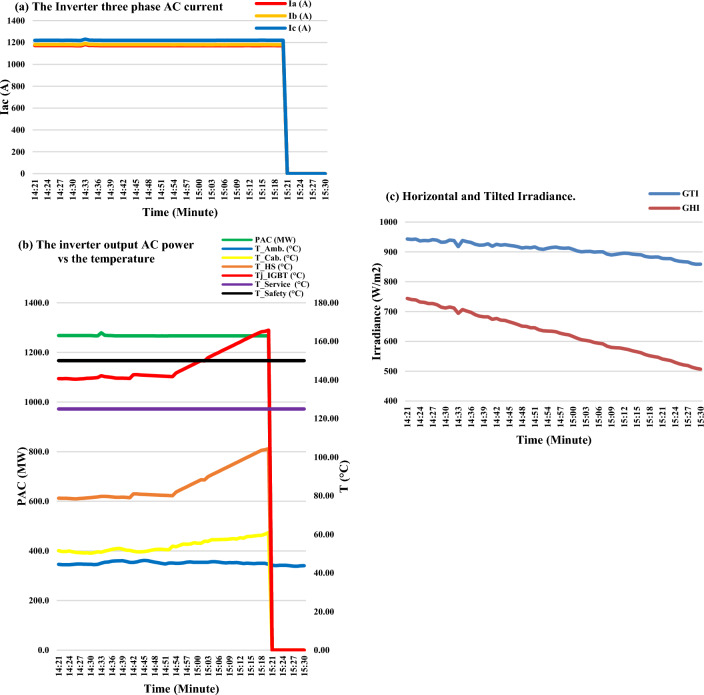
The operational data representation during the period from 02:21 PM to 03:30 PM.

Although the three-phase current had slightly decreased as the GTI radiation curve started to slightly decrease as shown in Fig. 7a and c respectively, the heatsink temperature gradually increased up to 105 °C as shown in Fig. 7b. The IGBT junction temperature jumped over the safety limit at the time of 03:00 PM. At the time of 03:21 PM, the IGBT was thermally broken down, as the IGBT operated for more than 20 min within a junction temperature higher than the safety limit. After that, the inverter completely burnt out.

### Investigation failure causes analysis

Based on the data representation shown in Figs. 6 and 7, the detected symptoms are as follows:

- Disturbance in radiation curve as shown in Fig. 6.
- Disturbance in the heatsink temperature, then high and gradually increasing in the last 20 min before failure as shown in Fig. 6.
- High junction temperature which started gradually increasing in the last 20 min before failure as shown in Fig. 7b.
- Slightly unbalanced in three-phase inverter output current as shown in Fig. 7a.

Based on the detected symptoms, the following scenarios can be expected as failure cause:

- Scenario (1): On the incident day, the SCADA didn’t record grid disturbances. So, the failure scenario due to the grid disturbances is excluded.
- Scenario (2): Before the incident by 2 h, there are no radiation disturbances which indicate the risks due to PV faults are low and excluded from failure scenarios.
- Scenario (3): There are clear symptoms observed which are described by increasing in heatsink as shown in Fig. 6 and increasing IGBT junction temperature as shown in Fig. 7b. Hereby, the cabinet temperature is increased as shown in Fig. 6, but the ambient temperature is approximately constant as shown in Fig. 6, which proves that there is no effect for the ambient temperature in the increase of both heatsink temperature and ambient temperature. On the other side, both the AC current and AC power are approximately constant during this period which started at 02:52 PM to 03:21 PM, so the possibility of considering the failure scenario due to the cooling fan malfunction is high. Hence, the investigation is highly recommended for this scenario and RCA is performed as follows:

As per the manufacturer’s datasheet, the maximum operating temperature is 80 °C and the insulation of the fan motor is class F. As per IEC 60034-1, the maximum safe operating temperature for the induction motor with class F insulation is 155 °C as mentioned in Table 1^30^. It is calculated as the relation given in Eq. (5). According to IEC 60034-1, the ambient temperature is equal to 40 °C^31^.

**Table 1:** The cooling fan technical specifications.

Insulation Class	T_max.OP_ (°C)	T_max.D_ @ loading 100% (°C)	T_max.D_ @ loading 115% (°C)	T_hotspot_
A	105	60	70	+ 5
B	130	80	90	+ 10
F	155	105	115	+ 10
H	180	125	Not Defined	+ 15

5 $${T}_{Max. Op.}={T}_{amb. M.}+ {T}_{Max. D}+ {T}_{hotspot}$$

**Figure 8:**
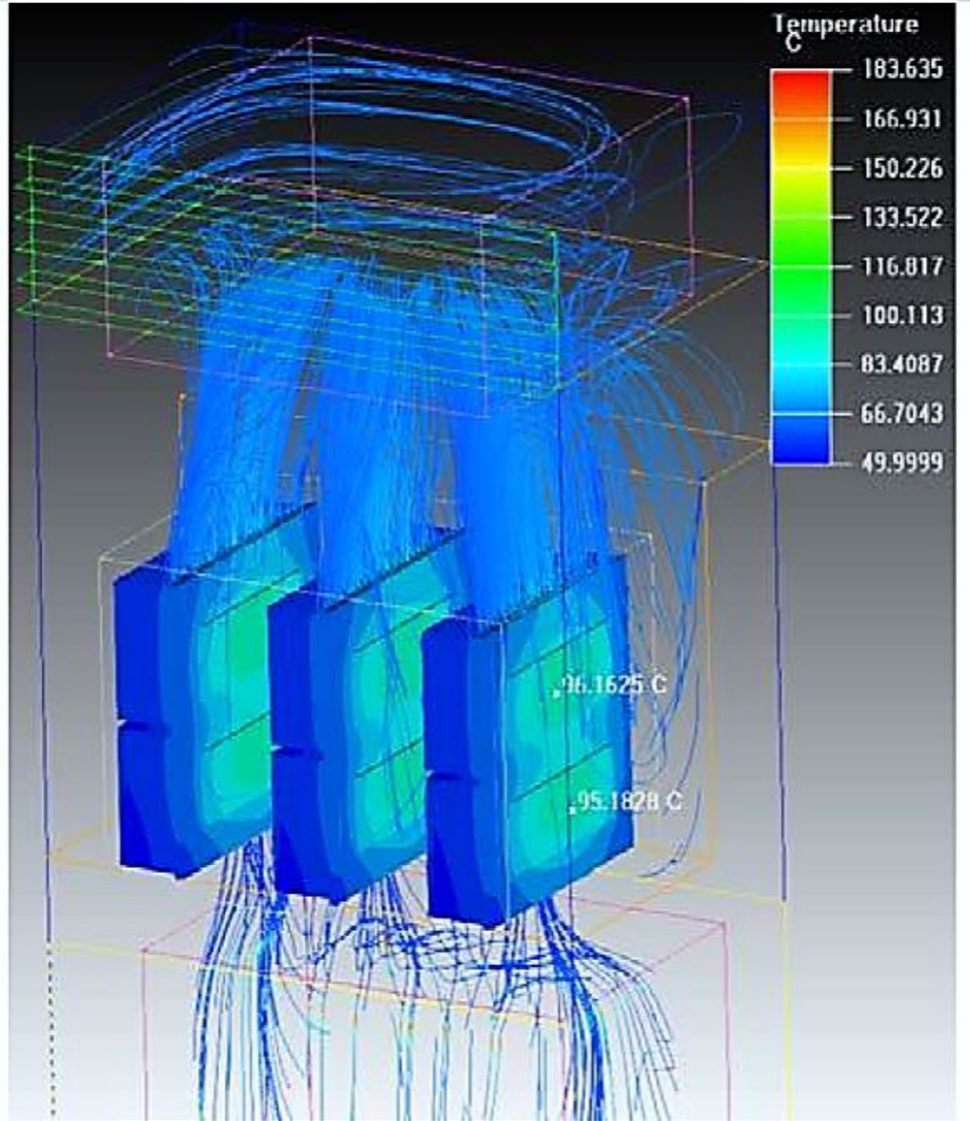
The thermal representation of power electronic switches and the medium around the cooling fan.

The ambient medium around the cooling fan is the cabinet air. So, the motor of the cooling fan operated at an ambient temperature equal to the cabinet temperature (T_amb.M._ = T__Cab_). During the summer period, the cabinet temperature ranges from 55 to 65 °C which refers to the high maximum operating temperature for the cooling fan motor. The thermal simulation of the IGBT cooling system is shown in Fig. 8, The ambient temperature of the surrounding medium exceeds 83 °C. Hereby, the maximum operating temperature for the motor of the cooling fan will exceed its maximum limit. By substituting in Eq. (5), if T_amb.M._ is equal to 83°C, then T_max.Op._ = 83 + 105 + 10 = 198 °C. Any induction motor has a safety factor for transient operation. It is equal to 115% of the full load and the corresponding maximum operating temperature for the motor in the transient conditions will equal to 165°C (Insulation Class F).

**Figure 9:**
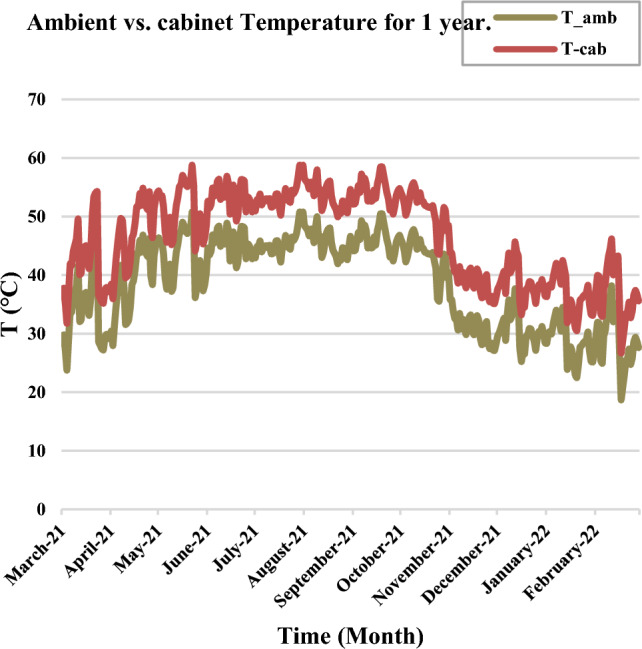
The ambient and cabinet temperature for one year.

Because of the high surrounding temperature, the motor of the cooling fan operated at a high ambient temperature for long periods during the summer session as shown in Fig. 9. So, the winding insulation suffered from gradual degradation. By the time the winding resistance is going to fail, then the thermal breakdown occurs for the motor of the cooling fan. If the insulation of motor winding is failed, the SCADA system can’t monitor this failure. Because the start/stop feedback of the cooling fan is given by the contactor contacts.

As a result of the cooling fan failure, the IGBT thermal resistance was increased and led to the heatsink temperature increasing which is considered an obvious symptom for the IGBT junction temperature increasing. Then the IGBT failure occurred due to heavy thermal stress.

### Detecting the signature of the IGBT thermal stress on the voltage balance of the DC link capacitor

**Figure 10:**
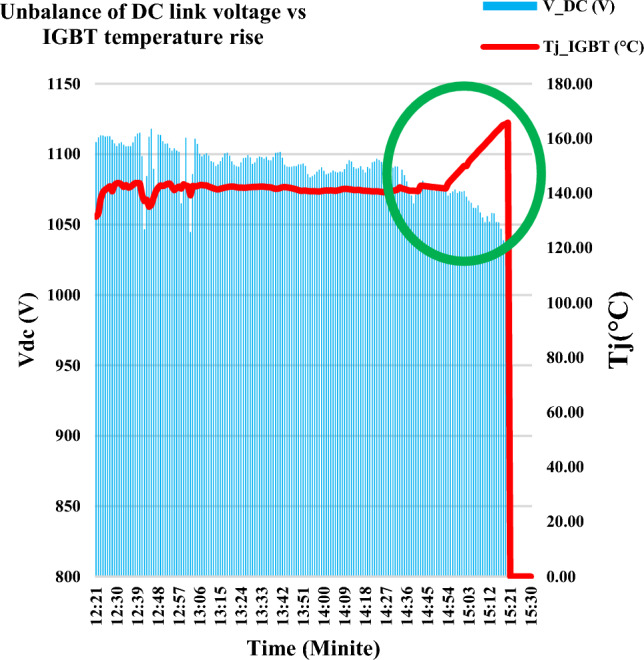
DC link voltage vs IGBT junction temperature.

During the period from 02:21 PM to 03:20 PM, although the radiation GTI curve is approximately constant, there are several drops in the DC link voltage as marked inside the green circle in Fig. 10. This voltage drop might occur as there is unbalance in the DC link capacitors voltage of the NPC inverter due to overheating of one or more of the IGBT. This event is matched with the fault signature which was investigated in^32^ and classified as Temperature Signature Analysis (TSA).

### The expected short circuit patterns for the DC link capacitors that lead to firing the inverter

**Figure 11:**
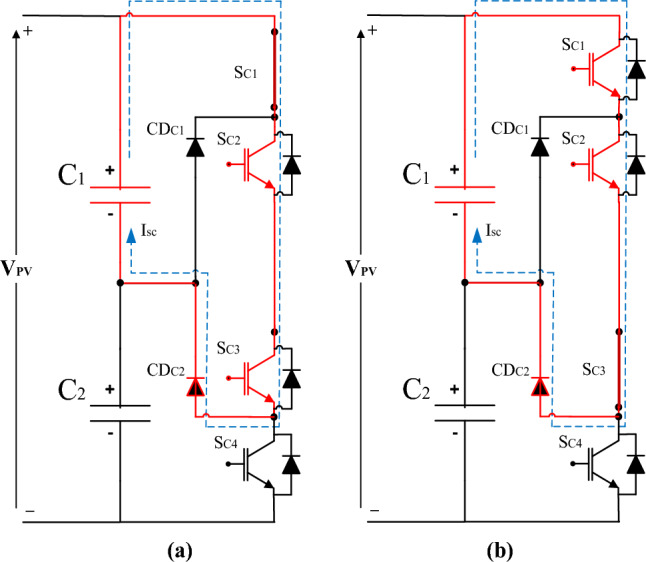
The upper DC link capacitor (C_1_) suffers from a short circuit pattern.

Due to the thermal stress, an internal short circuit fault occurs and the IGBT is turning on continuously. Then the DC link capacitors suffer from the short circuit path. The expected short circuit paths are as follows:

If the switch SC1 or the switch SC3 is turned on, the upper DC link capacitor (C_1_) suffers from the short circuit pattern as shown in Fig. 11a or b respectively.

**Figure 12:**
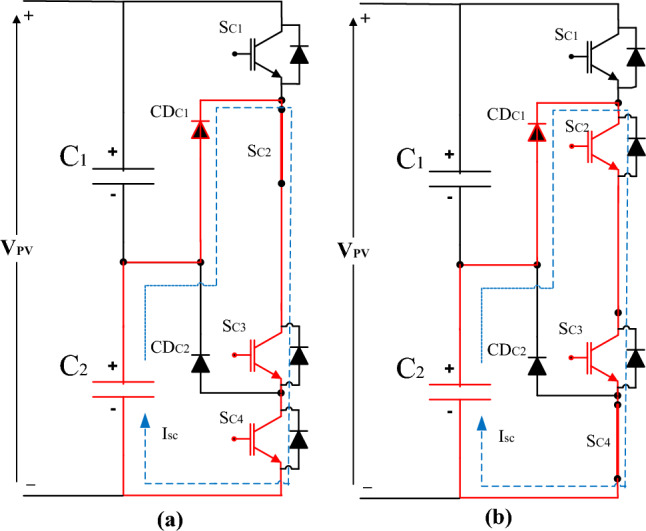
The lower DC link capacitor (C_2_) suffers from a short circuit pattern.

If the switch SC2 or the switch SC4 is turned on, the lower DC link capacitor (C_2_) suffers from the short circuit pattern as shown in Fig. 12a or b respectively.

**Figure 13:**
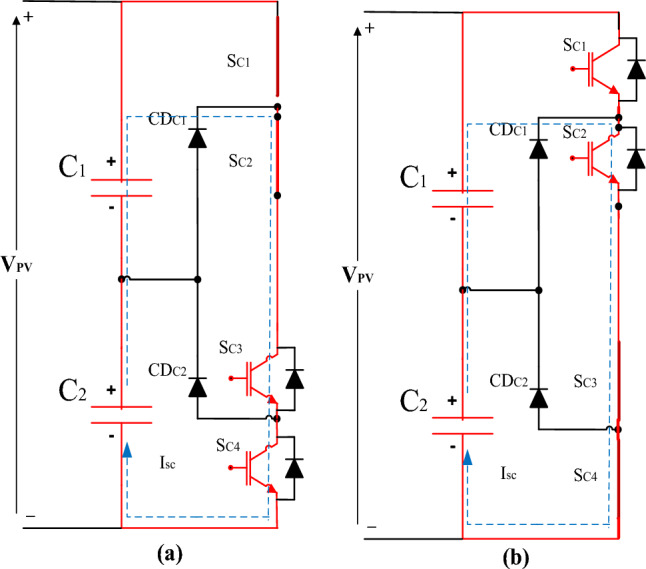
The complete DC link capacitor (C_1_&C_2_) suffers from a short circuit pattern.

If the switches SC1&SC2 or the switches SC3&SC4 are turned on simultaneously, the complete DC link capacitor (C_1_&C_2_) suffers from the short circuit pattern as shown in Fig. 13a or b respectively.

## Discussion

The previous investigation consists of the following stages:

- The data gathering stage which includes the collection of all the available monitored data for the charred inverter in addition to the questionnaires with the Operation and Maintenance (O&M) team to illustrate the event and identify the approximate status before the incident.
- The data processing stage which includes the methodologies for deducting the unmeasured parameter such as the IGBT junction temperature that is used to detect the thermal stress which occurred inside the IGBT before the failure.
- The data representation stage which visualized the operational data of the charred inverter to deduce symptoms indicate to the fault signatures that denote the thread-tip for the RCA process.
- The stage of verification for the expected scenario which maybe lead to the failure cause. As discussed in the section of the investigation of the failure caused in this paper that the unmonitored stopping of the cooling fan led to increasing in the IGBT junction temperature, then the IGBT suffered from a short circuit fault led to a complete thermal breakdown.
- The stage of detecting the secondary consequences of the IGBT thermal breakdown due to the short circuit fault. Hereby the explosion of the DC link capacitor led to high sparks which is considered the fire ignition in the inverter container, then the inverter is completely charred.

## Results

The investigation outcomes include three results as follows:

- The 1st result is detecting the signature of the IGBT thermal stress on the voltage balance of the DC link capacitor. There are several drops in the DC link voltage as marked inside the green circle in Fig. 10. This voltage drop occurred as there is unbalance in the DC link capacitors voltage of the NPC inverter due to overheating of one or more of the IGBT.
- The 2nd result is verifying a scenario for the cause of the IGBT failure by implementing a technical mathematical model based on the detected symptoms that denote the fault signature which is considered the thread-tip for detecting the failure cause. The junction temperature is calculated using the mathematical model as per Eq. (4). The junction inferred junction temperature is shown in the red curve of Fig. 7b.
- The 3rd result is the simulating scenario for the interpretation of a DC link capacitors explosion due to the short circuit fault. The DC link capacitors suffered from a short circuit path which led to the DC link capacitor explosion, then heavy sparks were produced and led to enough fire to burn the inverter container completely. The expected short circuit pattern for the upper DC link capacitor (C_1_) is shown in Fig. 11a or b. While the expected short circuit pattern for the lower DC (C_2_) link capacitor is shown in Fig. 12a or b. Also, the expected short circuit pattern for the complete DC link capacitor (C_1_&C_2_) is shown in Fig. 13a or b.
- The advantages of this investigation are that focuses on the real operational data (per one-minute interval) and extracts symptoms for a logical RCA scenario which is validated mathematically based on scientific principles. While the previous investigations that are reviewed in^17^, focused on the instantaneous continuous data (micro-second data) which used to represent the parameter waveforms. These previous models assumed the fault and validated the case through a simulation model and practical model in a lab experimental setup. While the investigated work in this paper considered the backward direction by starting from the real operational data gathering for the charred inverter, then simulated the case and validated that mathematically through logic RCA scenario.
- The disadvantage of this methodology that it is needs more field investigation and questionnaire with the operation and maintenance team which means that the possibility of human error is high if someone feedback with wrong information in addition to the detected symptoms from the operational data representation may lead to multiple RCA scenarios and can’t identify which of them is the real one. Also, analysis of big data of thousands per minute value is not easy and needs great efforts to be performed to get some symptoms to be considered as thread-tip denotes the failure cause of the grid-tie PV inverter.

### Comparison between the applied method and the classical method

The applied methodology can be briefly compared with the classical methods as reviewed in^17^ as recorded in Table 2.

**Table 2:** A brief comparison between the DD and the OD.

Item	Classical method based on the Instantaneous data measured per μs to ns	Applied method based on the operation data measured per minute
Measurement	Continuous Measurement	Discontinuous Measurement
Sampling	Instantaneous	Discrete
Sample Time	μs (Micro-second) to (Nano-second) ns	Minute
Hardware	Embedded computerized system	SCADA system
Storing Memory Size	Very High	Low
System Cost	High	Low
Complexity	High	Low
Cyber security	Complex	Easy
Suitability	Transient fault	Steady State fault
Effective response time	Short	Long
Validity	In case of instantaneous data recorded in μs to ns	Valid for operational data measures per minute interval
Advantages	Accurate and instantaneous monitoring	Easy and cheap system
Disadvantages	Complex, expensive system and limited to the lab stage	Can’t identify the transient fault

### Highlights recommendations for manufacturer and O&M team stages

The investigated work in this paper based on the discussion and results recommends the following factor to be considered for the manufacturing stage which can be considered during the design and fabrication:

- The inverter monitoring system shall have an algorithm to calculate the IGBT temperature from the measured parameters.
- The cooling fan shall be monitored through a current sensor in addition to contactor contact.
- The micro-second interval data shall be transferred to the SCADA system memory frequently to avoid its loss due to any failure.
- The inverter monitoring system shall represent the measured operational data into curves and compare many inverters in the same plant through the SCADA system to detect any observation or abnormal conditions during the inverter’s operational life cycle.

The investigated work in this paper based on the discussion and results recommends the following factor to be considered for the Operation and Maintenance (O&M) stage:

- Represent the inverter’s operational data of all the Mega-scale PV power plants and make a comparison for all the inverter input and output parameters. Then detect the difference or abnormal performance. The O&M team shall take fast action by derating the inverter output power or isolating the inverter till finding an explanation for those abnormal conditions.
- Maintain the preventive maintenance as possible to maintain the inverter reliability as possible.
- Check the inverter cooling fan frequently to ensure its correct and good operation.

## Conclusions

The investigation in this paper is performed based on operation data analysis of the PV grid-connected inverter (central type) due to a real incident. The analysis methodology is based on mathematical calculation for the IGBT junction temperature using the measured heatsink temperature. The study concludes that after the IGBT failure occurred, it was a short circuit for a while and closed the terminals of the DC link capacitors. So, the DC link capacitors exploded and produced heavy sparks that led to enough fire to burn the inverter container completely.

On the incident day, the SCADA didn’t record grid disturbances. So, the failure scenario due to the grid disturbances is excluded. Also, before the incident by 2 h, there is no radiation disturbances. This indicates that the risks due to PV faults are low. So, the possibility of failure due to the PV faults is excluded.

As a result of the cooling fan failure, the IGBT heatsink temperature increased. This indicates to increase in IGBT junction temperature as plotted and discussed previously. The heavy thermal stress on the IGBTs led to the thermal breakdown. Due to the thermal stress, an internal short circuit fault occurs and the IGBT is turning on continuously. Then the DC link capacitors suffer from the short circuit path, then the capacitors explode. Hereby, several heavy sparks are generated and lead to the firing of the inverter.

The advantages of this investigation are that focuses on the real operational data (per one-minute interval) and extracts symptoms for a logical RCA scenario which is validated mathematically based on the scientific principles. While the previous investigations that are reviewed in^17^, focused on the instantaneous continuous data (micro-second data) which used to represent the parameter waveforms. These previous models assumed the fault and validated the case through a simulation model and practical model in a lab experimental setup. While the investigated work in this paper considered the backward direction by starting from the real operational data gathering for the charred inverter, then simulated the case and validated that mathematically through logic RCA scenario.

The disadvantage of this methodology that it is needs more field investigation and questionnaire with the operation and maintenance team which means that the possibility of human error is high if someone feedback with wrong information in addition to the detected symptoms from the operational data representation may lead to multiple RCA scenarios and can’t identify which of them is the real one. Also, analysis of big data of thousands per minute value is not easy and needs great efforts to be performed to get some symptoms to be considered as thread-tip denotes the failure cause of the grid-tie PV inverter.

## Future work

The future work for the proposed methodology considers a simulation model which can be built through MATLAB Simulink software to simulate the inverter of the case of study in the same conditions of the real profile (irradiance and temperature) to detect and monitor the performance and detect a prediction technique to denote the fault which may lead to the IGBT failure, so the inverter can be protected from the risk of completely burning.

## Data Availability

The data are gathered from the PV plant SCADA system in addition to the questionnaires by the O&M team. It will be provided upon request by the Correspondence author.

## List of symbols

I_a_: RMS current of phase a
I_b_: RMS current of phase b
I_c_: RMS current of phase c
P_AC_: The AC active power (Inverter Output)
T_Amb_: The ambient temperature
T__Cab_: The inverter cabinet temperature
T__HS_: The heatsink temperature
GHI: The horizontal global irradiance
GTI: The tilted global irradiance
IGBT: Isolated Gate Bipolar Transistor
FWD: Free Wheeling Diode
${P}_{IGBT\_Cond}$: The IGBT conduction power loss
${P}_{IGBT\_Sw}$: The IGBT switching power loss
${P}_{IGBT\_Loss}$: The IGBT total power loss
${V}_{CE}$: The drop voltage between the IGBT Emitter–Collector
${I}_{IGBT\_av}$: IGBT average current
${I}_{IGBT\_rms}$: The IGBT RMS current
${r}_{CE}$ or *R*_*s*_: The IGBT ON resistance
${K}_{g}$: The correction factor = 1.1–1.2
${f}_{s}$: The switching frequency
${V}_{\mathrm{op}}$: The IGBT operating blocking voltage
${I}_{\mathrm{op}}$: The IGBT operating current
${V}_{Nom}$: The IGBT nominal blocking voltage
${I}_{Nom}$: The IGBT nominal current
${E}_{\mathrm{on}}$: The energy loss during turns ON
${E}_{off}$: The energy loss during turns OFF
${T}_{J\_IGBT}$: The IGBT junction temperature
$${T}_{HS}$$: Heatsink temperature
${R}_{T\_th-JC}$: The IGBT thermal resistance (Junction to Case)
${R}_{D\_th-JC}$: The FWD thermal resistance (Junction to Case)
${R}_{C\_th-TA}$: The thermal resistance (Heatsink to Ambient)
${R}_{eq\_th(J-Cas)}$: The IGBT & FWD & SG equivalent thermal resistance
*T*_*_Safety*_: The temperature safety limit
*T*_*_Service*_: The temperature service limit
*T*_*max.OP*_: The maximum operating temperature of the cooling fan motor
*T*_*max.D*_: The maximum designed temperature of the cooling fan motor
*T*_*hotspot*_: The hotspot temperature in the fan motor winding
RCA: Root Cause Analysis
